# Metformin Enhances Excitatory Synaptic Transmission onto Hippocampal CA1 Pyramidal Neurons

**DOI:** 10.3390/brainsci10100706

**Published:** 2020-10-04

**Authors:** Wen-Bing Chen, Jiang Chen, Zi-Yang Liu, Bin Luo, Tian Zhou, Er-Kang Fei

**Affiliations:** 1 School of Life Sciences, Nanchang University, Nanchang 330031, China; cwb100405@163.com (W.-B.C.); ncuchenjiang@163.com (J.C.); nculzy2020@163.com (Z.-Y.L.); ncusskluo@163.com (B.L.); 2 Institute of Life Science, Nanchang University, Nanchang 330031, China; 3 School of Basic Medical Sciences, Nanchang University, Nanchang 330031, China; zhoutian@ncu.edu.cn

**Keywords:** metformin, glutamate release, neuronal excitability, hippocampal, pyramidal neurons

## Abstract

Metformin (Met) is a first-line drug for type 2 diabetes mellitus (T2DM). Numerous studies have shown that Met exerts beneficial effects on a variety of neurological disorders, including Alzheimer’s disease (AD), Parkinson’s disease (PD) and Huntington’s disease (HD). However, it is still largely unclear how Met acts on neurons. Here, by treating acute hippocampal slices with Met (1 μM and 10 μM) and recording synaptic transmission as well as neuronal excitability of CA1 pyramidal neurons, we found that Met treatments significantly increased the frequency of miniature excitatory postsynaptic currents (mEPSCs), but not amplitude. Neither frequency nor amplitude of miniature inhibitory postsynaptic currents (mIPSCs) were changed with Met treatments. Analysis of paired-pulse ratios (PPR) demonstrates that enhanced presynaptic glutamate release from terminals innervating CA1 hippocampal pyramidal neurons, while excitability of CA1 pyramidal neurons was not altered. Our results suggest that Met preferentially increases glutamatergic rather than GABAergic transmission in hippocampal CA1, providing a new insight on how Met acts on neurons.

## 1. Introduction

The biguanide metformin (Met) has long been used as a first-line drug for type 2 diabetes mellitus (T2DM) therapy. Met lowers the plasma glucose level through inhibiting hepatic glucose production and enhancing insulin sensitivity [1]. At the cellular level, Met inhibits the mitochondrial respiratory chain complex I and activates AMP (adenosine monophosphate)-activated protein kinase complex (AMPK) [2,3]. Met can also target other organelles, such as lysosome, or act in an AMPK-independent manner [4,5,6].

Although Met is the mainstay of therapy for T2DM, it has also been indicated that Met may have effects in some neurological disorders, such as neurodevelopmental disorders, neurodegenerative diseases and neuropsychiatric disorders. In a fragile X syndrome (FXS) mouse model, Met treatment ameliorated social deficit, repetitive behavior and abnormal dendritic spine morphology and exaggerated long-term depression (LTD) [7]. Furthermore, improvements in language and cognitive behaviors were observed in a small sample size of Met-treated FXS patients [8,9,10]. In some Alzheimer’s disease (AD) mice models, Met attenuated amyloid plaque deposition and improved learning and memory [11,12,13,14]. Met also showed neuroprotective effects on dopaminergic neurons in 1-methyl-4-phenyl-1,2,3,6-tetrahydropyridine (MPTP)-modeled mice of Parkinson’s disease (PD) [15,16]. Moreover, Met has been found to rescue the early brain changes and abnormal behaviors in Huntington’s disease (HD)-modeled in mice [17]. Furthermore, Met was suggested to benefit depression by studies in both animals [18,19] and T2DM patients with depression [20]. These findings indicate the beneficial effects of Met on brain functions.

It has been shown that Met may exert anti-inflammatory, anti-oxidative and anti-apoptotic effects on neurological disorders [21]. The neuroprotective effects of Met can be achieved by actions on mitochondria, the main target organelle of Met [22]. Met can also regulate brain metabolism and homeostasis for the improvement of brain functions [23,24,25]. Neurons are the primary signaling cells and the functional unit in the brain. The communication between neurons, also called synaptic transmission, is crucial to brain functions. However, it remains unclear how Met acts on the neurons and synapses. To uncover the role of Met on synaptic transmission, here, we examined the effects of Met on glutamatergic and GABAergic neurotransmission in hippocampal slices from mice.

## 2. Materials and Methods

### 2.1. Animals

Male C57BJ/6 mice of 5–6 weeks of age were used. The mice were housed in a room of 22 °C in a 12 h light/dark cycle with free access to food and water ad libitum. All experiment procedures were approved by the Institutional Animal Care and Use Committee of Nanchang University.

### 2.2. Reagents

Met was purchased from Sigma-Aldrich, and 6-cyano-7-nitroquinoxaline-2,3-dione (CNQX), L-2-amino-5-phosphonopentanoic acid (DL-AP5) and bicuculline were purchased from Tocris Bioscience (Bristol, UK). Tetrodotoxin (TTX) was purchased from the Fisheries Research Institute of Hebei province (Hebei, China).

### 2.3. Slice Preparation

Mice (6–7-week-old) were anesthetized with isoflurane and subjected to cardiac perfusion with an ice-cold oxygenated (95% O_2_/5% CO_2_) choline chloride-based cutting solution, containing: 120 mM choline chloride, 2.5 mM KCl, 7 mM MgCl_2_, 0.5 mM CaCl_2_, 1.25 mM NaH_2_PO_4_, 5 mM sodium ascorbate, 3 mM sodium pyruvate, 26 mM NaHCO_3_, and 25 mM glucose. The mice were rapidly decapitated and their brains were removed quickly and placed in a cutting solution. Hippocampal slices of 300 µm thickness were prepared with VT1000S Vibratome (Leica Microsystems) [26]. Slices were transferred to storage chamber containing a cutting solution at 34 °C for 15 min, then translocated to the incubator with artificial cerebrospinal fluid (ACSF) (124 mM NaCl, 2.5 mM KCl, 2 mM MgSO_4_, 2.5 mM CaCl_2_, 1.25 mM NaH_2_PO_4_, 26 mM NaHCO_3_, and 10 mM glucose) at room temperature (25 ± 1 °C) for at least 1 hr before recording.

### 2.4. Electrophysiological Recording

Slices were exposed to Met for 1 hr in an individual incubation chamber, then placed in a recording chamber superfused (2 mL/min) with ACSF at 32–34 °C. Pyramidal neurons in CA1 were visualized with infrared optics using an upright fixed microscope equipped with a 40X water-immersion lens (FN1, Nikon) and a CCD (Charge-Coupled Device) monochrome video camera (IR-1000, DAGE-MTI). Patch pipettes (resistance of 3–5 MΩ) were prepared by a horizontal pipette puller (P-1000; Sutter Instruments).

For miniature excitatory postsynaptic current (mEPSC) recording, pyramidal neurons were held at −70 mV in the presence of 20 µM bicuculline and 1 µM TTX, with the pipette solution containing: 125 mM K-gluconate, 5 mM KCl, 10 mM HEPES, 0.2 mM EGTA, 1 mM MgCl_2_, 4 mM Mg-ATP, 0.3 mM Na-GTP and 10 mM phosphocreatine (pH 7.35, 290 mOsm). For miniature inhibitory postsynaptic current (mIPSC) recording, pyramidal neurons were held at −70 mV in the presence of 20 µM CNQX, 50 µM DL-AP5 and 1 µM TTX, with the pipette solution containing: 130 mM CsCl, 10 mM HEPES, 0.2 mM EGTA, 1 mM MgCl_2_, 4 mM Mg-ATP, 0.3 mM Na-GTP, mM 10 phosphocreatine and 5 mM QX314 (pH 7.35, 290 mOsm) [27].

For paired-pulse ratio recording, EPSCs were evoked by stimulating the Schaffer collaterals (SC)-CA1 pathway at a holding potential of −70 mV in the presence of 20 µM bicuculline, with the pipette solution containing: 125 mM Cs-methanesulfonate, 5 mM CsCl, 10 mM HEPES, 0.2 mM EGTA, 1 mM MgCl_2_, 4 mM Mg-ATP, 0.3 mM Na-GTP, 10 mM phosphocreatine and 5 mM QX314 (pH 7.35, 290 mOsm). The first evoked EPSC was adjusted with amplitude between 100 and 150 pA. The ratio was defined as the fraction of EPSC2/EPSC1 amplitudes.

To measure neuronal intrinsic excitability, recording pipettes were filled with internal solutions containing 125 mM K-gluconate, 5 mM KCl, 10 mM HEPES, 0.2 mM EGTA, 1 mM MgCl_2_, 4 mM Mg-ATP, 0.3 mM Na-GTP and 10 mM phosphocreatine (pH 7.35, 290 mOsm), the current clamp mode was performed and pulsed depolarization currents with increasing amplitude at steps of 50 pA were injected [28]. Furthermore, 20 µM CNQX, 50 µM DL-AP5 and 20 µM bicuculline were added to the perfusion buffer to block excitability and inhibitory neurotransmission.

Data were acquired using the MultiClamp 700B amplifier and 1550A digitizer (Molecular Devices). Series resistance was monitored throughout the experiments and cells included in analysis were of resistance <20 MΩ. Neurons would be rejected if membrane potentials were more positive than −60 mV, if the ratio of Rin to Rs <5 or if series resistance fluctuated >20% of initial values. Data were filtered at 3 kHz and sampled at 10 kHz.

### 2.5. Statistical Analysis

Data were analyzed with GraphPad Prism software. For comparison between three groups, one-way ANOVA was used, followed by Tukey’s post hoc multiple comparison test. For analysis of three groups at multiple time points, two-way ANOVA was used, followed by Bonferroni’s post hoc multiple comparison test. The mEPSCs and mIPSCs were manually analyzed by Mini Analysis (Synaptosoft). The paired-pulse ratio and action potential numbers were analyzed by clampfit (Molecular Devices). Cumulative probability curves for inter-event interval and amplitude of mEPSC/mIPSC were analyzed using the Kolmogorov–Smirnov test. Data were presented as mean ± SEM. *p*-value < 0.05 was considered statistically significant.

## 3. Results

### 3.1. Met Markedly Enhances Glutamatergic Transmission in the CA1 Pyramidal Neurons

**Figure 1:**
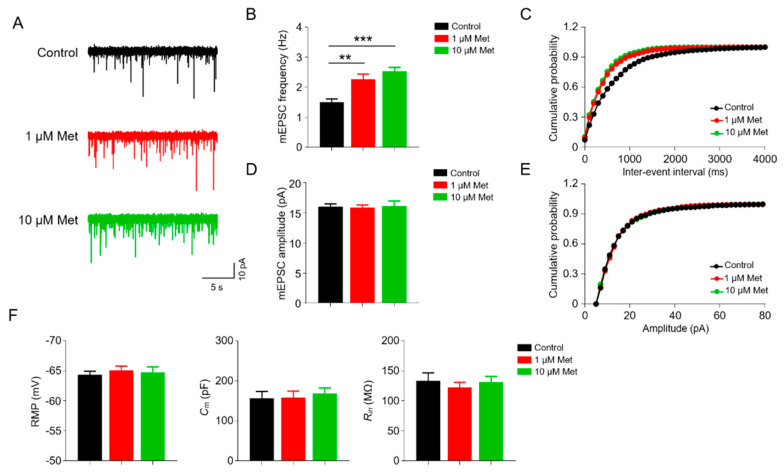
Increased excitability transmission in metformin (Met)-treated hippocampal slices. (**A**) Representative traces showing miniature excitatory postsynaptic current (mEPSC) in CA1 pyramidal neurons incubated with 0, 1, 10 μM Met. Scale bar = 5 s, 10 pA. (**B**) Increased mEPSC frequency of pyramidal neurons in Met-treated hippocampal slices. *n* = 12–13 neurons from four mice per group. F(2,35) = 14.53, *p* < 0.0001; control (1.51 ± 0.11 Hz) versus 1 μM Met (2.27 ± 0.17 Hz), *p* = 0.0011; control (1.51 ± 0.11 Hz) versus 10 μM Met (2.53 ± 0.13 Hz), *p* < 0.0001; 1 μM Met (2.27 ± 0.17 Hz) versus 10 μM Met (2.53 ± 0.13 Hz), *p* = 0.4090; one-way ANOVA followed by Tukey’s post hoc test. (**C**) Cumulative probability curves for mEPSC inter-event interval. Control versus 1 μM Met, *p* = 0.0324; control versus 10 μM Met, *p* = 0.0003; 1 μM Met versus 10 μM Met, *p* = 0.1744; Kolmogorov–Smirnov test. (**D**) No difference in mEPSC amplitude of pyramidal neurons among groups. *n* = 12–13 neurons from four mice per group. F(2,35) = 0.0475, *p* = 0.9537; control (16.06 ± 0.44 pA) versus 1 μM Met (15.91 ± 0.41 pA), *p* = 0.9817; control (16.06 ± 0.44 pA) versus 10 μM Met (16.16 ± 0.83 pA), *p* = 0.9912; 1 μM Met (15.91 ± 0.41 pA) versus 10 μM Met (16.16 ± 0.83 pA), *p* = 0.9499; one-way ANOVA followed by Tukey’s post hoc test. (**E**) Cumulative probability curves for mEPSC amplitude. Control versus 1 μM Met, *p* = 0.7307; control versus 10 μM Met, *p* = 0.9998; 1 μM Met versus 10 μM Met, *p* = 0.9844; Kolmogorov–Smirnov test. (**F**) There was no difference in rest membrane potential (RMP), membrane capacitance and input resistance of pyramidal neurons between the control and Met treatment group; *n* = 12 ~ 13 neurons from three mice per group. Data are presented as mean ± SEM. ** *p* < 0.01, *** *p* < 0.001.

We first investigated the potential role of Met on glutamatergic transmission by performing whole-cell voltage clamp recordings on CA1 pyramidal neurons. Miniature excitatory postsynaptic currents (mEPSCs) were recorded, with the presence of bicuculline (antagonist of GABAA receptors) and tetrodotoxin (TTX, voltage-gated sodium channel blocker) blocking inhibitory neurotransmission and action potential (AP), respectively (Figure 1). mEPSC frequencies were dramatically increased in slices treated with Met at concentrations of 1 μM and 10 μM (F(2,35) = 14.53, *p* < 0.0001, one-way ANOVA; control (1.51 ± 0.11 Hz) versus 1 μM Met (2.27 ± 0.17 Hz), *p* = 0.0011; control (1.51 ± 0.11 Hz) versus 10 μM Met (2.53 ± 0.13 Hz), *p* < 0.0001; *n* = 12–13 neurons from four mice per group) (Figure 1A,B). In addition, no difference in frequency was observed between the 1 μM Met and 10 μM Met groups (Figure 1A,B). As a result, both 1 μM and 10 μM Met treatment considerably left-shifted the cumulative probability curve of mEPSC inter-event interval (control versus 1 μM Met, *p* = 0.0324; control versus 10 μM Met, *p* = 0.0003; Kolmogorov–Smirnov test) (Figure 1C). In contrast, neither the 1 μM nor 10 μM Met treatments showed influence on mEPSC amplitude compared with the control group (F(2,35) = 0.0475, *p* = 0.9537; one-way ANOVA; control (16.06 ± 0.44 pA) versus 1 μM Met (15.91 ± 0.41 pA), *p* = 0.9817; control (16.06 ± 0.44 pA) versus 10 μM Met (16.16 ± 0.83 pA), *p* = 0.9912) (Figure 1D). Accordingly, there was no significant effect of 1 μM or 10 μM Met on the cumulative probability curve of mEPSC amplitude (control versus 1 μM Met, *p* = 0.7307; control versus 10 μM Met, *p* = 0.9998; Kolmogorov–Smirnov test) (Figure 1E). Moreover, there was no difference in passive membrane properties, including rest membrane potential (RMP), membrane capacitance and input resistance of pyramidal neurons between the control and the Met treatment groups. Taken together, these results suggest that Met enhances the glutamatergic transmission onto CA1 pyramidal neurons.

### 3.2. Met Has No Effect on the GABAergic Transmission in the CA1 Pyramidal Neurons

**Figure 2:**
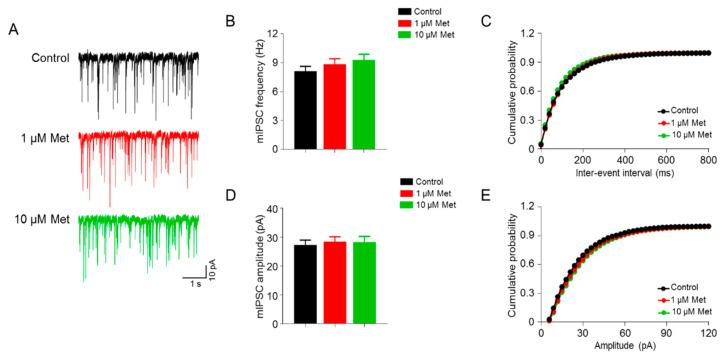
Inhibitory transmission was not altered in Met-treated hippocampal slices. (**A**) Representative traces showing miniature inhibitory postsynaptic currents (mIPSCs) in CA1 pyramidal neurons incubated with 0, 1 and 10 μM Met. Scale bar = 1 s, 10 pA. (**B**) No difference in mIPSC frequency of pyramidal neurons among groups. *n* = 17–18 neurons from four mice per group. F(2,49) = 1.085, *p* = 0.3458, control (8.12 ± 0.51 Hz) versus 1 μM Met (8.86 ± 0.57 Hz), *p* = 0.6155; control (8.12 ± 0.51 Hz) versus 10 μM Met (9.28 ± 0.60 Hz), *p* =0.3220; 1 μM Met (8.86 ± 0.57 Hz) versus 10 μM Met (9.28 ± 0.60 Hz), *p* = 0.8569; one-way ANOVA followed by Tukey’s post hoc test. (**C**) Cumulative probability curves for mIPSC inter-event interval. Control versus 1 μM Met, *p* = 0.9206; control versus 10 μM Met, *p* = 0.5886; 1 μM Met versus 10 μM Met, *p* = 0.9898; Kolmogorov–Smirnov test. (**D**) No difference in mIPSC amplitude of pyramidal neurons among groups. *n* = 17–18 neurons from four mice per group. F(2,49) = 0.1204, *p* = 0.8869, control (27.29 ± 1.70 pA) versus 1 μM Met (28.43 ± 1.67 pA), *p* = 0.8921; control (27.29 ± 1.70 Hz) versus 10 μM Met (28.28 ± 1.99 pA), *p* = 0.9206; 1 μM Met (28.43 ± 1.67 pA) versus 10 μM Met (28.28 ± 1.99 pA), *p* = 0.9978; one-way ANOVA followed by Tukey’s post hoc test. (**E**) Cumulative probability curves for mIPSC amplitude. Control versus 1 μM Met, *p* = 0.7453; control versus 10 μM Met, *p* = 0.5562; 1 μM Met versus 10 μM Met, *p* > 0.9999; Kolmogorov–Smirnov test. Data are presented as mean ± SEM.

We next explored the influence of Met on the GABAergic transmission onto CA1 pyramidal neurons. Neurons were clamped at −70 mV and the miniature inhibitory postsynaptic currents (mIPSCs) were recorded in the presence of 20 µM CNQX, 50 µM DL-AP5 (antagonists of α-amino-3-hydroxyl-5-methyl-4-isoxazole-propionate (AMPA) and N-methyl-D-aspartate (NMDA) receptors, respectively) and 1 μM TTX, which blocks excitatory neurotransmission and APs (Figure 2). As shown in Figure 2A,B, neither 1 μM nor 10 μM Met changed mIPSC frequency (F(2,49) = 1.085, *p* = 0.3458, one-way ANOVA; control (8.12 ± 0.51 Hz) versus 1 μM Met (8.86 ± 0.57 Hz), *p* = 0.6155; control (8.12 ± 0.51 Hz) versus 10 μM Met (9.28 ± 0.60 Hz), *p* = 0.3220; *n* = 17–18 neurons from four mice per group). In line with this, the cumulative probability curves of mEPSC inter-event interval were similar between the control and Met treatment (control versus 1 μM Met, *p* = 0.9206; control versus 10 μM Met, *p* = 0.5886; Kolmogorov–Smirnov test) (Figure 2C). Furthermore, no significant changes in the mean value and cumulative probability curve of mIPSC amplitude were found in the Met treatment group compared with control group (mean value: F(2,49) = 0.1204, *p* = 0.8869, one-way ANOVA; control (27.29 ± 1.70 pA) versus 1 μM Met (28.43 ± 1.67 pA), *p* = 0.8921; control (27.29 ± 1.70 Hz) versus 10 μM Met (28.28 ± 1.99 pA), *p* = 0.9206; cumulative probability curve: control versus 1 μM Met, *p* = 0.7453; control versus 10 μM Met, *p* = 0.5562; Kolmogorov–Smirnov test) (Figure 2D,E). Altogether, these data suggest that Met has no effect on GABAergic transmission onto CA1 pyramidal neurons.

### 3.3. Met Increases Glutamate Release from Presynaptic Terminals

**Figure 3:**
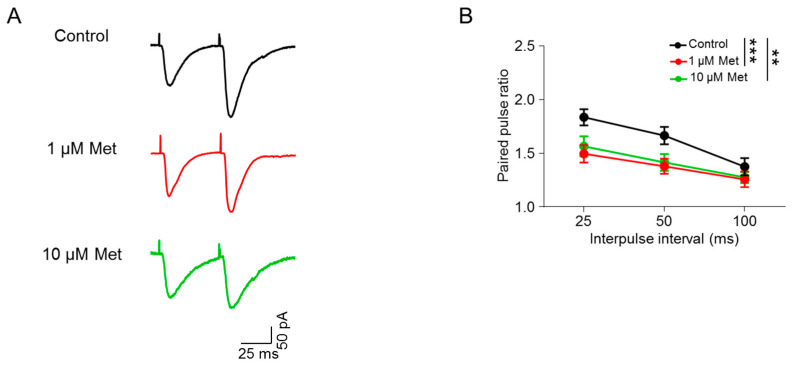
Met increases glutamate release in CA1 hippocampal pyramidal neurons. (**A**) Representative traces of pair-pulse stimulation. Scale bar = 25 ms, 50 pA. (**B**) Paired-pulse responses (PPRs) plotted against inter-stimulate intervals. *n* = 9–11 neurons from three mice per group. Drug treatment: control versus 1 μM Met, F(1,51) = 15.54, *p* = 0.0002; control versus 10 μM Met, F(1,57) = 10.72, *p* = 0.0018; 1 μM Met versus 10 μM Met, F(1,54) = 0.4391, *p* = 0.5104; two-way ANOVA followed by Bonferroni’s post hoc test. Data are presented as mean ± SEM, ** *p* < 0.01, *** *p* < 0.001.

The increased frequency of mEPSCs suggests increased glutamatergic transmission but we cannot tell the specific cause (e.g., the increased release probability or the number of presynaptic terminals). To address this question, we measured the paired-pulse responses of evoked EPSCs (eEPSCs) at different intervals in CA1 pyramidal neurons. The electric stimuli were delivered through a bipolar electrode placed in the Schaffer collaterals (SC)-CA1 pathway. Neurons were held at −70 mV in the presence of 20 µM bicuculline to block GABAergic transmission. The paired-pulse ratio (PPR) was calculated by the fraction of EPSC2 by EPSC1 amplitudes. As shown in Figure 3, we found that PPRs were significantly decreased in 1 μM and 10 μM Met treatment groups compared with the control group (control versus 1 μM Met, F(1,51) = 15.54, *p* = 0.0002; control versus 10 μM Met, F(1,57) = 10.72, *p* = 0.0018; two-way ANOVA; *n* = 9–11 neurons from three mice per group), indicating that Met enhances glutamate release probability from presynaptic terminals that innervate hippocampal CA1 pyramidal neurons.

### 3.4. Met Does Not Alter Intrinsic Excitability in CA1 Pyramidal Neurons

**Figure 4:**
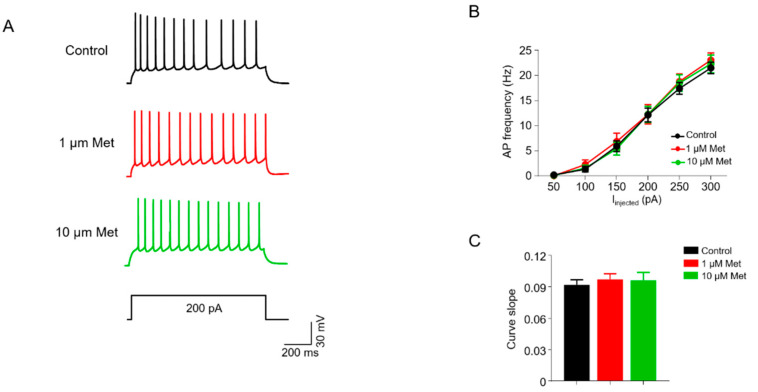
Met does not affect neuronal excitability in CA1 hippocampal pyramidal neurons. (**A**) Representative traces of spikes evoked by injecting 200 pA depolarizing currents. Scale bar = 200 ms, 30 mV. (**B**) Firing rate plotted against increasing injected depolarizing currents. Control, *n* = 14 neurons/4 mice; 1 μM Met, *n* = 15 neurons/5 mice; 10 μM Met, *n* = 16 neurons/5 mice; F(2,252) = 0.5951, *p* = 0.5523; two-way ANOVA. (**C**) Comparisons of the curve slope in B. F(2,42) = 0.1984, *p* = 0.8208, control (0.092 ± 0.005) versus 1 μM Met (0.097 ± 0.005), *p* = 0.8278; control (0.092 ± 0.005) versus 10 μM Met (0.096 ± 0.008), *p* = 0.8704; 1 μM Met (0.097 ± 0.005) versus 10 μM Met (0.096 ± 0.008), *p* = 0.9950; one-way ANOVA followed by Tukey’s post hoc test. Data are presented as mean ± SEM.

We next examined whether Met affects the neuronal intrinsic excitability of CA1 pyramidal neurons. The APs of pyramidal neurons were recorded in a whole-cell current clamp configuration by injecting depolarization currents. CNQX, DL-AP5 and bicuculline were added into a bath solution to block excitatory and inhibitory neurotransmission. We measured the AP numbers in response to injected currents of incremental levels and the firing frequency curve of pyramidal neurons were similar between the three groups (F(2,252) = 0.5951, *p* = 0.5523; two-way ANOVA; control, *n* = 14 neurons/4 mice; 1 μM Met, *n* = 15 neurons/5 mice; 10 μM Met, *n* = 16 neurons/5 mice) (Figure 4A,B). Consistently, no differences among groups were observed in the slope of fitted curves plotting the spike number against the current strength (F(2,42) = 0.1984, *p* = 0.8208, one-way ANOVA; control (0.092 ± 0.005) versus 1 μM Met (0.097 ± 0.005), *p* = 0.8278; control (0.092 ± 0.005) versus 10 μM Met (0.096 ± 0.008), *p* = 0.8704) (Figure 4C). These results suggest that the intrinsic excitability of pyramidal neurons is not influenced by Met treatment.

## 4. Discussion

In the present study, we demonstrated that Met, a first-line drug for T2DM, enhances glutamate release onto hippocampal CA1 pyramidal neurons. We treated acute hippocampal slices with Met at 1 μM or 10 μM concentrations and found that Met treatments at both concentrations increased mEPSC frequency, but not amplitude. Met treatments did not alter mIPSC frequency and amplitude. Moreover, presynaptic glutamate release probability, which was measured by PPRs, was increased upon Met treatment. However, Met treatments did not affect neuronal excitability in hippocampal CA1 pyramidal neurons. These results present evidence that Met enhances glutamatergic transmission, but not GABAergic transmission, onto hippocampal CA1 pyramidal neurons.

Neurotransmission is fundamental for brain functions, and defects in neurotransmission are implicated in many neurological disorders, such as AD, FXS, depression and so on. Met has been suggested to be effective in these disorders [8,9,10,29,30]. Although Met exerts its glucose level-lowering effects in the peripheral system, it has been reported that Met can cross the brain–blood barrier rapidly [31]. The hippocampus is a key brain region for memory and cognition. Neuronal activity and synaptic transmission within the hippocampus are critical for these functions. The hippocampus is also one of the major brain regions affected by AD, in which hippocampal synaptic transmission is impaired [32]. Met has been demonstrated to ameliorate synaptic, memory and cognitive deficits in rodent models of AD [12,33,34,35]. However, there are few reports about Met effects on synaptic transmission. Interestingly, we treated acute hippocampal slices with Met and found that mEPSC frequency, but not mIPSC frequency, was increased upon Met treatment (Figure 1; Figure 2). These data suggest that Met can preferentially increase glutamatergic rather than GABAergic transmission, which may contribute to the beneficial effects of Met on AD and other related disorders of the brain.

Either enhanced glutamate release or increased number of functional synapses could lead to the elevation of mEPSC frequency [36]. Thus, enhanced mEPSC frequency of hippocampal pyramidal neurons upon Met treatment (Figure 1) may be due to the increased presynaptic glutamate release. In support of this notion, the PPR of Met-treated hippocampal pyramidal neurons was indeed decreased (Figure 3). It seems like Met selectively regulates glutamatergic transmission. The frequency and amplitude of mIPSCs were not changed in hippocampal CA1 pyramidal neurons upon Met treatment (Figure 2). In peripheral cells, uptake of Met is mediated by the organic cation transporters OCT1 and OCT3 [37,38,39]. OCT3 is also found to be expressed in neurons [40,41,42]. Little is known about the expression pattern of Met transporters in the brains. There is a possibility that Met transporters are enriched only in excitatory neurons, thus contributing to the preference of Met in glutamatergic transmission. However, this needs further examination. Within cells, there are AMPK-dependent and -independent pathways for the action of Met. During synaptic activation, AMPK is required for maintaining energy levels [43]. It has been reported that food deprivation increases AMPK-dependent presynaptic glutamate release onto Agouti-related protein (AGRP) neurons [44]. AMPK has three subunits (α, β and γ), and AMPKα is abundant in hippocampal neurons [45]. Met treatment activates AMPK in the hippocampus of mice [46]. This pathway of Met on glutamatergic transmission needs further confirmation by treatment of AMPK inhibitors. Alternatively, Met can also act in an AMPK-independent pathway, through which Met may regulate presynaptic glutamate release machinery. Moreover, Met has neuroprotective effects in aging-related neurodegenerative diseases, such as AD and PD, and can improve the cognition of aged mice [47,48]. In the future, the effects of Met on synaptic transmission in aged mice should be investigated.

As mentioned above, functional synapse numbers can also affect mEPSC frequency. Next, spine density and morphology could be examined in primary hippocampal neuronal cultures treated with Met. Besides, silencing of synapses can contribute to the decreased mEPSC frequency and AMPA receptor turnover at the postsynaptic membrane regulates glutamatergic synapse silencing [49]. This raises the possibility that Met may selectively regulate trafficking of the AMPA receptor but not the GABA receptor at the postsynaptic area, which deserves to be examined in the future.

## 5. Conclusions

Taken together, our study, for the first time, suggested that Met preferentially enhances glutamate transmission onto hippocampal CA1 pyramidal neurons, which provides a new insight into the mechanism of the beneficial effect of Met on neurons.

## Data Availability

All data generated or analyzed during this study are included in this published article.
